# Permanent draft genome sequence of the probiotic strain *Propionibacterium freudenreichii* CIRM-BIA 129 (ITG P20)

**DOI:** 10.1186/s40793-015-0120-z

**Published:** 2016-01-14

**Authors:** Hélène Falentin, Stéphanie-Marie Deutsch, Valentin Loux, Amal Hammani, Julien Buratti, Sandrine Parayre, Victoria Chuat, Valérie Barbe, Jean-Marc Aury, Gwenaël Jan, Yves Le Loir

**Affiliations:** INRA, UMR 1253, Science et Technologie du Lait et de l’Oeuf, 35000 Rennes, France; AGROCAMPUS OUEST, UMR1253, UMR Science et Technologie du Lait et de l’Oeuf, 35000 Rennes, France; INRA, UR1077 Unité Mathématique Informatique et Génome, Jouy-en-Josas, France; CEA Genoscope CNRS and université d’Evry, 91 006 Evry, France

**Keywords:** GRAS, QPS, probiotic, anti-inflammatory, immunomodulation, surface proteins

## Abstract

*Propionibacterium freudenreichii* belongs to the class *Actinobacteria* (Gram positive with a high GC content). This “Generally Recognized As Safe” (GRAS) species is traditionally used as (i) a starter for Swiss-type cheeses where it is responsible for holes and aroma production, (ii) a vitamin B12 and propionic acid producer in white biotechnologies, and (iii) a probiotic for use in humans and animals because of its bifidogenic and anti-inflammatory properties. Until now, only strain CIRM-BIA1T had been sequenced, annotated and become publicly available. Strain CIRM-BIA129 (commercially available as ITG P20) has considerable anti-inflammatory potential. Its gene content was compared to that of CIRM-BIA1 T. This strain contains 2384 genes including 1 ribosomal operon, 45 tRNA and 30 pseudogenes.

## Introduction

*Propionibacterium freudenreichii* belongs to the class of *Actinobacteria* (Gram positive bacteria with a high GC content). This ‘Generally Recognized As Safe’ species is traditionally used as (i) a starter for Swiss-type cheeses where it is responsible for holes and aroma production (ii) a vitamin B12 and propionic acid producer in white biotechnologies, and (iii) a probiotic for use in humans and animals because of its bifidogenic properties (enhancing intestinal transit). A recent screening of 23 strains belonging to this species revealed the considerable anti-inflammatory properties of strain CIRM-BIA129 (ITG-P20) [1]. Its anti-inflammatory potential is superior to that of the previously sequenced CIRM-BIA1 T strain. CIRM-BIA129 (ITG-P20) is currently used to make cheese at an industrial scale and indeed is consumed in large quantities because one gram of Swiss-type cheese contains at least 10^9^ CFU. The genetic basis for the anti-inflammatory properties of *P. freudenreichii* are still poorly understood. Sequencing CIRM-BIA129 (ITG-P20) would enable investigation of the genomic determinants responsible for the important anti-inflammatory properties of this strain. To our knowledge, CIRM-BIA129 is the second strain in this species to be sequenced, annotated and made publicly available.

## Organism information

### Classification and features

**Fig. 1:**
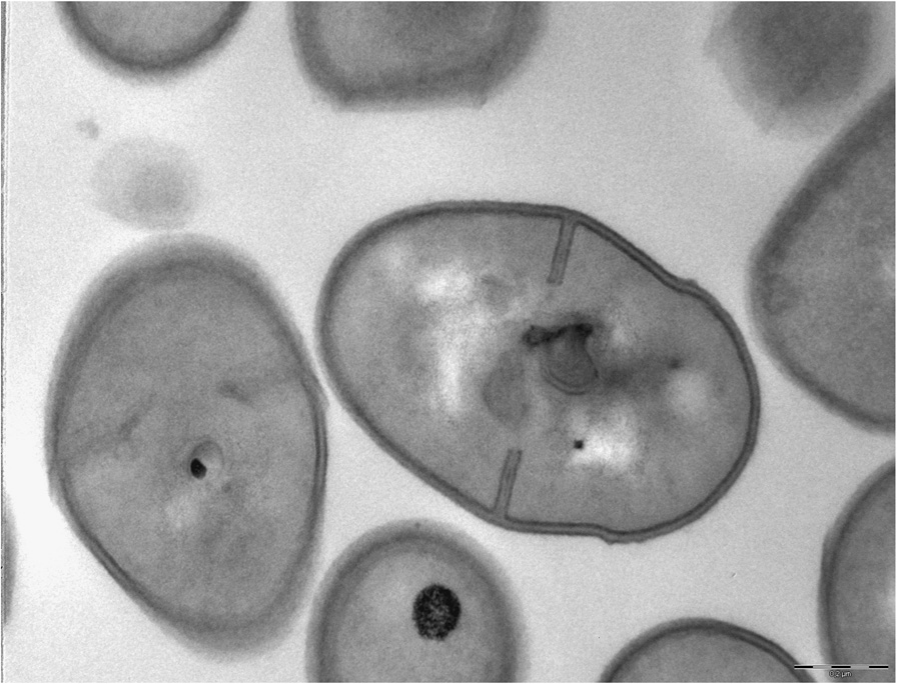
Transmission electron microscopy pictures of liquid-grown cultures of CIRM-BIA129 (ITG P20)

**Fig. 2:**
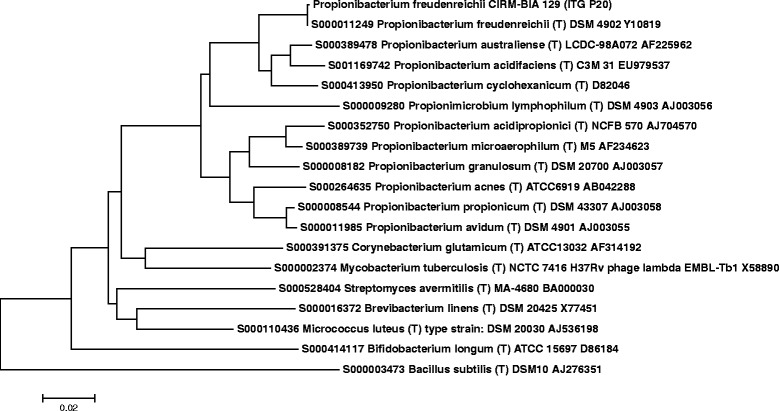
The evolutionary history of the strain was inferred using the Neighbor-Joining method [12]. The optimal tree with a sum of branch lengths = 0.80 is shown. The tree is drawn to scale, with branch lengths in the same units as those of the evolutionary distances used to infer the phylogenetic tree. The evolutionary distances were computed using the Maximum Composite Likelihood method [13] and are in the units of the number of base substitutions per site. The analysis involved 18 nucleotide sequences. All positions containing gaps and missing data were eliminated. There were a total of 1376 positions in the final dataset. Evolutionary analyses were performed under MEGA5 [14]

*P. freudenreichii* CIRM-BIA129 (ITG P20) cells are Gram-positive, microaerophilic, pleiomorphic (coccoid to rod shape forming ‘Chinese characters’) bacillae (1.0–1.5 μm × 0.5–0.8 μm wide) forming creamy-white colonies on YEL agar plates (Table 1). Cells grow at the bottom of liquid medium tubes (to escape oxygen) and tend to clot in liquid culture at the beginning of the stationary phase. Transmission electron microscopy pictures of liquid-grown cultures revealed a thick cell wall (Fig. 1) made of peptidoglycan. No exopolysaccharides were observed at the surface of the bacteria, unlike what is seen in numerous strains of *P. freudenreichii**,* including the type strain CIRM-BIA1 T (Fig. 2).

**Table 1:** Classification and general features of *P. freudenreichii* CIRM-BIA129 (ITG-P20) according to MIGS guidelines and the Catalogue of Life database

MIGS ID	Property	Term	Evidence code (a)
	Classification	Domain *Bacteria*	TAS [15]
		Phylum *Actinobacteria*	TAS [16, 17]
		Class *Actinobacteria*	TAS [16, 17]
		Order *Actinomycetales*	TAS [16]
		Family *Propionibacteriaceae*	TAS [18]
		Genus *Propionibacterium*	TAS [19]
		Species *Propionibacterium freundenreichii*	TAS [20]
		Strain CIRM-BIA 129 alias ITG P20	NAS
	Gram staining	Positive	NAS
	Cell shape	Pleiomorph (coccoids to rods)	NAS
	Motility	Non-motile	NAS
	Sporulation	Non-sporulating	NAS
	Temperature range	Not tested	
	Temperature optimum	32 °C	NAS
	pH range; Optimum	Not tested	
	Carbon source	glycerol, erythritol, L-arabinose, adonitol, galactose, D-glucose, D-fructose, D-mannose, inositol, arbutine, esculine, lactose, lactate and gluconate	IDA
MIGS-6	Habitat	Unknown	
MIGS-6.3	Salinity	Tolerate up to 1 M NaCl	IDA
MIGS-22	Oxygen requirement	Microaerophilic	NAS
MIGS-15	Biotic relationship	Free-living	NAS
MIGS-14	Pathogenicity	Non-pathogen, GRAS, QPS	NAS
MIGS-4	Geographic location	Not reported	
MIGS-5	Sample collection time	Not reported	
MIGS-4.1	Latitude	Not reported	
MIGS-4.2	Longitude	Not reported	
MIGS-4.3	Depth	Not reported	
MIGS-4.4	Altitude	Not reported	

(a) Evidence codes – IDA: Inferred from Direct Assay; TAS: Traceable Author Statement (i.e., a direct report exists in the literature); NAS: Non-traceable Author Statement (i.e., not directly observed for the living, isolated sample, but based on a generally accepted property for the species, or anecdotal evidence). These evidence codes are those of the Gene Ontology project [21]

A BLASTn of the 16S sequence of type strain CIRM-BIA1 T against the contigs of CIRM-BIA129 (ITG-P20) confirmed the affiliation of this strain to the species (100 % identity, 100 % coverage).

Representative genomic 16S rRNA sequences of the strains were compared with those of other type strains belonging to *Actinobacteria* present in the Ribosomal Database Project. (Figure 2) shows the phylogenetic tree.

CIRM-BIA129 (ITG P20) was found to be able to utilize glycerol, erythritol, L-arabinose, adonitol, galactose, D-glucose, D-fructose, D-mannose, inositol, arbutine, esculine, lactose, lactate and gluconate as carbon sources according to the gallery-API results.

CIRM-BIA129 (ITG P20) was shown to grow with up to 1 M NaCl in a chemically defined medium in presence of osmoprotectant (G. Jan, personal communication).

## Genome sequencing information

### Genome project history

*Propionibacterium freudenreichii* CIRM-BIA 129 (ITG P20) genome was sequenced to obtain information regarding mechanism(s) or molecule(s) responsible for anti-inflammatory properties of the strain. Project information and associated MIGS are shown in Table 2.

**Table 2:** Project information

MIGS ID	Property	Term
MIGS-31	Finishing quality	Improved high quality draft
MIGS-28	Libraries used	300 bp-insert Illumina library
MIGS-29	Sequencing platforms	36 nucleotide paired-end sequencing (Illumina genome analyser II).
MIGS-31.2	Fold coverage	681x
MIGS-30	Assemblers	Velvet version 1.1.03 k-mer
MIGS-32	Gene calling method	Show program
	Locus Tag	PFCIRM129
	EMBL ID	CCBE010000001-CCBE010000111
	EMBL Date of Release	20-Jun-2014
	BIOPROJECT	PRJEB4826
MIGS-13	Source Material Identifier	CIRM-BIA 129
	Project relevance	Probiotic, anti-inflammatory

### Growth conditions and genomic DNA preparation

The *P. freudenreichii* strain CIRM-BIA129 (ITG-P20), isolated from Emmental cheese by Actalia Dairy Products (Institut Technique du Gruyère, Actalia, Rennes, France), was provided by the CIRM-BIA Biological Resource Centre (Centre International de Ressources Microbiennes-Bactéries d’Intérêt Alimentaire, INRA, Rennes, France). It was cultivated at 30 °C without shaking in YEL [2]. Growth was monitored spectrophotometrically at 650 nm, as well as by counting colony-forming units in YEL medium containing 1.5 % agar.

A cell pellet (equivalent to 2 × 10^10^ CFU) was obtained by centrifugation for 10 min at 5000 × *g* from a one-day exponentional phase culture of CIRM-BIA129 (ITG P20). DNA was extracted using the Blood & Cell Culture DNA Midi Kit (Qiagen) according to the manufacturer’s recommendations with the following modifications. Briefly, complete bacterial lysis was obtained by adding 220 mg of lysozyme powder (Qbiogene) to 3.5 ml of B1 buffer (Qiagen) followed by 2.5 h of incubation at 37 °C. High molecular weight genomic DNA was purified by gravity flow and anion exchange chromatography, eluted in 5 ml QF buffer (Qiagen) and precipitated with 3.5 ml of isopropanol. DNA was collected by centrifugation for 10 min at 4 °C and 15000 *g* and then air dried. DNA was resuspended in 100 μL TE 1X buffer at pH8 (Sigma) for two hours at 55 °C. DNA integrity was checked on an 0.8 % agarose gel. The OD_260/280nm_ was 1.9.

### Genome sequencing and assembly

The genome of the CIRM-BIA129 (ITG-P20) strain was obtained using a whole-genome strategy based on Illumina 36 nucleotide paired-end sequencing (Illumina genome analyser II) (Table 2). After filtration and trimming, the number of reads was downsized to 50 million of 34.8 bases mean read length, representing a 681-fold genome coverage. The reads were assembled using Velvet version 1.1.03 [3] with a k-mer size of 31. The assembly resulted in 59 scaffolds of a length greater than 1 kb, with an N50 of 123 kb.

### Genome annotation

Automatic and manual annotations were carried out with the AGMIAL platform [4], using *P. freudenreichii* CIRM-BIA 1 T as the reference [5]. Sub-cellular localization was predicted using SurfG+ [6], a software specific to sub-cellular prediction in Gram-positive bacteria. A comparison of the predicted proteome of CIRM-BIA129 with that of the sequenced type strain CIRM-BIA1 was performed with Koriblast software (Korilog, France) with its default parameters. Genes present in CIRM-BIA129 and not in the type strain CIRM-BIA1 were searched by tblastn of the CIRM-BIA129 predicted proteome against the complete genome of CIRM-BIA1. No hit proteins were specific to CIRM-BIA129. In order to search for genomic islands specific to CIRM-BIA129, a blastp of the CIRM-BIA129 proteome against the CIRM-BIA1 proteome was performed. Proteins filtered for a ‘hit coverage’ of less than 75 % and those corresponding to locus-tags colocalized along the chromosome were attributed to genomic islands. To search for probable pseudogenes in CIRM-BIA1, a blastp analysis of CIRM-BIA1 protein against CIRM-BIA129 proteins filtered on a ‘hit coverage’ of less than 75 % was performed. Among them, the corresponding genes were declared as pseudogenes in the event of two adjacent genes having the same ‘best hit’. S-layer homology domains inside the proteins were sought from the SLH.hmm pattern using hmmer with default parameters.

## Genome properties

The genome was found to be composed of 111 contigs for a total size of 2,588,969 bp (67.3 % GC content). The contig length was encompassed between 158 bp and 179,656 bp with an average at 23,324 bp. The N50 is 46,655 bp. One ribosomal operon (containing one 16S rRNA gene and one 23S rRNA gene) was present, on contig 24, A total of 2354 complete genes were predicted, 2,307 of them protein-coding genes and 45 tRNA encoding genes. Thirty genes were pseudogenes. According to the COG results, 1,236 protein coding genes were assigned to a putative function, the remainder being annotated as hypothetical proteins. The properties and statistics of the genome are summarized in (Tables 3 and 4).

**Table 3:** Nucleotide content and gene count levels of the genome

Attribute	Value	% of total^(a)^
Genome size (bp)	2,588969	100
DNA coding (bp)	2,209128	85.3
DNA G + C (bp)	1,741869	67.3
DNA scaffolds	59	100
Total genes	2,388	100
Protein-coding genes	2,307	96.6
RNA genes	3	0.2
Pseudo genes	30	1.2
Genes in internal clusters	Not reported	Not reported
Genes with function prediction	1190	49.8
Genes assigned to COGs	1,325	55.5
Genes with Pfam domains	1148	48.1
Genes with signal peptides	247	10.3
Genes with transmembrane helices	521	21.8
CRISPR repeats	Not reported	Not reported

(a)The total is based on the size of the genome in base pairs

**Table 4:** Number of genes associated with 25 general COG functional categories (2337 proteins)

Code	Value	% age	Description
J	116	4.96	Translation, ribosomal structure and biogenesis
A	1	0.04	RNA processing and modification
K	83	3.55	Transcription
L	76	3.25	Replication and repair
B	0	0	Chromatin structure and dynamics
D	14	0.60	Cell cycle control and mitosis
V	30	1.28	Defence mechanisms
T	32	1.37	Signal transduction mechanisms
M	58	2.48	Cell wall/membrane biogenesis
N	0	0	Cell motility
U	16	0.68	Intracellular trafficking and secretion
O	51	2.18	Post-translational modification, protein turnover, chaperone functions
C	88	3.77	Energy production and conversion
G	119	5.09	Carbohydrate metabolism and transport
E	121	5.18	Amino acid metabolism and transport
F	47	2.01	Nucleotide metabolism and transport
H	88	3.77	Coenzyme metabolism
I	38	1.63	Lipid metabolism
P	88	3.77	Inorganic ion transport and metabolism
Q	21	0.90	Secondary structure
R	149	6.38	General function prediction only
S	89	3.81	Function unknown
-	1012	43.3	Not in COG

The total is based on the total number of protein coding genes in the genome

## Insights into the genome sequence

### Surface proteins encoding genes

In a previous research study [7], the authors observed that the removal of surface-associated proteins led to a marked decrease in anti-inflammatory properties. Numerous studies dealing with the anti-inflammatory properties of food or probiotic bacteria have also identified the S-layer proteins or other surface compounds responsible for this trait [8, 9].

The S-layer proteins were found to be paracrystalline mono-layered assemblies of proteins which coat the surface of bacteria. S-layer proteins were associated with the cell wall via an SLH domain, with a cell wall polymer serving as the anchoring structure. This SLH domain comprised about 40–50 amino-acids, and could be found as one or more copies in the protein. Proteins other than S-layer proteins are known to be anchored by SLH domains to the cell wall of bacteria and are called S-layer associated proteins [10]. As *P. freudenreichii* CIRM-BIA129 is known for its anti-inflammatory properties [7, 11], it was necessary to determine the presence of protein sequences containing SLH domains. This search led to the identification of eight genes: *slpB* (PFCIRM129_00700) that contained five SLH domains; *slpE*, (PFCIRM129_05460) containing four SLH domains; *slpF* (PFCIRM129_01545), *slpG* (PFCIRM129_09890), *slpA* (PFCIRM129_09350) and *inlA* (PFCIRM129_12235) that contained three SLH domains at the C-terminal part, and *slh2* (PFCIRM129_03800) and *slpD* (PFCIRM129_11775), containing two SLH domains. Interestingly, CIRM-BIA129 (ITG-P20) did not present a typical, thick S-layer at its surface, so these genes most probably code for SLAPs.

### Comparison with the reference strain

A tblastn analysis revealed 77 new genes (no hit) that were not present in the type strain CIRM-BIA1. Most of them were of unknown function except for four, which encoded two transporters PFCIRM129_07475 (pseudogene) and PFCIRM129_08355, an amidohydrolase PFCIRM129_03910 and a transcription factor PFCIRM129_03595. The blastp results revealed three genomic islands that were present in CIRM-BIA129 but absent from CIRM-BIA1: (i) from PFCIRM129_09365 to 09660 encompassed by conjugal transfer protein gene PFCIRM_09215 and conjugative transfer gene complex protein PFCIRM129_09665, probably corresponding to an integrative conjugal element, (ii) from _10870 to _10910 comprising several relaxase genes and an exopolyphosphatase gene PFCIRM129_10900, and (iii) from PFCIRM129_10830 to 10905 containing three alpha, beta hydrolase genes (PFCIRM129_10860, 10890, 10895).

A blastp analysis revealed a gene encoding a ribulokinase PFCIRM129_07785 which was pseudogenized in CIRM-BIA129 but appeared to be functional in CIRM-BIA1.

By contrast, the glucokinase gene PFREUD_00950 involved in gluconate degradation was pseudogenized in CIRM-BIA1 but complete in CIRM-BIA129. This difference may explain the ability of CIRM-BIA129 to degrade gluconate. In the same way, *inlA* (containing three SLH domains, see above) was pseudogenized in CIRM-BIA1 but complete in CIRM-BIA129. This suggests a better ability to interact with eukaryotic cells, as internalin A was described as a bacterial adhesin.

## Conclusion

The genome of CIRM-BIA129 revealed new genes that had never previously been described in the species. Some of them, which probably encode surface exposed proteins, may be of considerable importance to the adaptation of the bacterium to the intestinal tract. Its ability to degrade gluconate may enable it to survive in the intestine where this sugar is abundant. Genes including the SLH domain may be candidates for the immune properties of the strain because SLH domains enable the protein to be anchored in the cell wall.

## Abbreviations

YEL: yeast extract lactate
